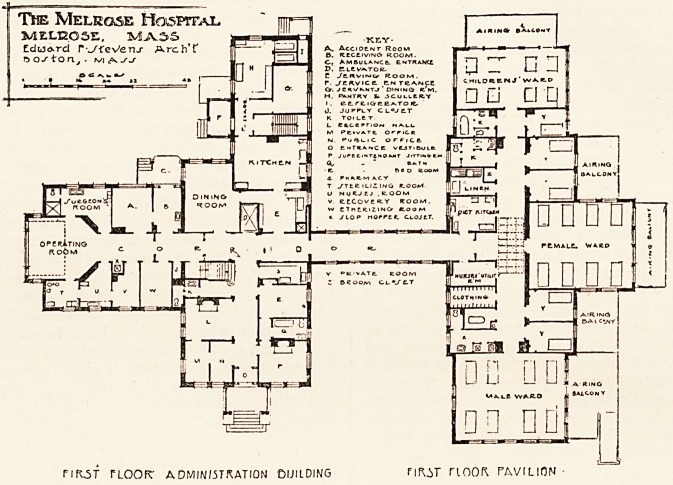# An American Cottage Hospital

**Published:** 1911-02-18

**Authors:** 


### AN AMERICAN COTTAGE HOSPITAL.

The plans of the proposed new hospital at Melrose, Mass., U.S.A., which have just been published by the Melrose Hospital Association, are interesting, as they show considerable modifications of the usual scheme adopted for small hospitals of this class—which really belong to the cottage hospital type. The perspective view of the proposed buildings reveals a dignified simple frontage, which is not over elaborately embellished with architectural addenda, while it impresses the observer with the fact that the building itself is, from an architectonic point of view, as excellent as it could be.

FIRST FLOOR ADMINISTRATION BUILDING FIRST FLOOR PAVILION

The ground plans of the first floor administration block and of the first floor of the pavilion, which we here reproduce, show clearly the simplicity of the general design.

The new buildings are to be as complete as any small hospital in the States. The plan just adopted by the trustees fixes the building as one of the semi-pavilion type, so arranged that the maximum amount of sun and air will be received in every room where it is needed. The cost is estimated at £15,000.

The administration and operating building are in one block, while the general wards are in the pavilion, the two being connected by a corridor.

In the main or administration building are arranged the offices, reception room, a suite of rooms for the superintendent, the kitchen for the entire hospital, nurses' and servants' dining-rooms, together with storerooms and refrigerator. On the north side of the administration building is the operating department, consisting of admitting room, accident room, main operating room, etherising and recovery rooms, doctors' rooms, sterilising and nurses' rooms, making a most complete department.

Connected by a corridor is the ward building or pavilion, which contains an eight-bed male ward, an eight-bed female ward, and eight-bed children's wards, together with five private rooms, a large diet kitchen, linen and clothing rooms, toilet and baths.

Each ward and private room is provided with an airing balcony, so that every patient can be wheeled directly to the open air with the least amount of effort.

In the second story of the administration building are arranged private rooms, a maternity ward, and a complete maternity department.

The nurses' quarters are temporarily to be in the second and third stories.

It is contemplated to erect the building either with concrete or with tile and concrete veneer, with tile or slate roof.

The committee has had the assistance of Mr. Edward F. Stevens, architect, a hospital specialist of Boston, whose years of experience in this line of work guarantees the very best results.

## Figures and Tables

**Figure f1:**